# Mitochondrial Metabolism in the Spotlight: Maintaining Balanced RNAP III Activity Ensures Cellular Homeostasis

**DOI:** 10.3390/ijms241914763

**Published:** 2023-09-29

**Authors:** Roza Szatkowska, Emil Furmanek, Andrzej M. Kierzek, Christian Ludwig, Malgorzata Adamczyk

**Affiliations:** 1 Laboratory of Systems and Synthetic Biology, Chair of Drugs and Cosmetics Biotechnology, Faculty of Chemistry, Warsaw University of Technology, Noakowskiego 3, 00-664 Warsaw, Poland; roza.pitruska@gmail.com (R.S.);; 2 Certara UK Limited, Sheffield S1 2BJ, UK; andrzej.kierzek@certara.com; 3 School of Biosciences and Medicine, University of Surrey, Guildford GU2 7XH, UK; 4 Institute of Metabolism and Systems Research, University of Birmingham, Birmingham B15 2TT, UK; c.ludwig@bham.ac.uk

**Keywords:** RNA polymerase III, RNAP III, TCA cycle, mitochondrial metabolism, MAF1, ^13^C flux, systems biology, growth rate

## Abstract

RNA polymerase III (RNAP III) holoenzyme activity and the processing of its products have been linked to several metabolic dysfunctions in lower and higher eukaryotes. Alterations in the activity of RNAP III-driven synthesis of non-coding RNA cause extensive changes in glucose metabolism. Increased RNAP III activity in the *S. cerevisiae maf1Δ* strain is lethal when grown on a non-fermentable carbon source. This lethal phenotype is suppressed by reducing tRNA synthesis. Neither the cause of the lack of growth nor the underlying molecular mechanism have been deciphered, and this area has been awaiting scientific explanation for a decade. Our previous proteomics data suggested mitochondrial dysfunction in the strain. Using model mutant strains *maf1Δ* (with increased tRNA abundance) and *rpc128-1007* (with reduced tRNA abundance), we collected data showing major changes in the TCA cycle metabolism of the mutants that explain the phenotypic observations. Based on ^13^C flux data and analysis of TCA enzyme activities, the present study identifies the flux constraints in the mitochondrial metabolic network. The lack of growth is associated with a decrease in TCA cycle activity and downregulation of the flux towards glutamate, aspartate and phosphoenolpyruvate (PEP), the metabolic intermediate feeding the gluconeogenic pathway. *rpc128-1007*, the strain that is unable to increase tRNA synthesis due to a mutation in the C128 subunit, has increased TCA cycle activity under non-fermentable conditions. To summarize, cells with non-optimal activity of RNAP III undergo substantial adaptation to a new metabolic state, which makes them vulnerable under specific growth conditions. Our results strongly suggest that balanced, non-coding RNA synthesis that is coupled to glucose signaling is a fundamental requirement to sustain a cell’s intracellular homeostasis and flexibility under changing growth conditions. The presented results provide insight into the possible role of RNAP III in the mitochondrial metabolism of other cell types.

## 1. Introduction

Mitochondria are considered a major source of energy production and a signaling hub [1]. When mitochondrial biological function is compromised, dysfunction in cellular homeostasis follows, leading to aging and disease. In recent years, the mechanism of action of RNA polymerase III (RNAP III) has been researched extensively but rarely in relation to metabolic homeostasis, nor specifically to mitochondrial metabolism [2]. Primarily, RNAP III is responsible for the synthesis of non-coding RNAs, including tRNA, 5S rRNA and U6 snRNA, 7SL RNA, the RNA component of ribonuclease P (RNase P), snoRNA, the RNA component RNase MRP, 7SL RNA, vault RNAs, Y RNAs, and SINE-encoded RNAs [3,4]. The less abundant RNAP III products are involved in diverse processes such as pre-mRNA splicing, rRNA processing, regulation of RNAP II transcription, macromolecular assembly and/or transport in metazoans and chromosomal DNA replication in human cells [5]. The activity of RNAP III is inhibited by an evolutionarily conserved regulator, the MAF1 protein, in response to intra- and extracellular signals such as a change in carbon source, entry into a stationary growth phase, DNA damage, oxidative stress, or cell treatment with rapamycin or chlorpromazine [6,7,8,9,10,11,12]. The MAF1 protein is the only negative regulator of RNAP III in *Saccharomyces cerevisiae* [6]. Consequently, MAF1-deficient cells (*maf1Δ*) show higher RNAP III activity, leading to increased levels of some classes of tRNAs and their precursors decoupled from environmental cues [2,13,14]. A changed ratio of tRNA:rRNA transcripts in these mutants has been reported. Moreover, low steady-state levels of 3′ extended tRNAs were detected in *maf1Δ* [14]. Apart from its function in the process of tRNA transcription, high RNAP III activity is negatively correlated with the activity of high-affinity glucose transporters and positively correlated with glycolytic pathway activity in *S. cerevisiae* [15,16]. Furthermore, the increased activity of RNAP III caused by the deletion of the MAF1 gene leads to growth defects on a non-fermentable carbon source at 37 °C, which has been linked to gluconeogenesis [17]. However, the hypothesis that downregulation of FBP1 transcriptional activity, which may lead to decreased fructose bisphosphatase levels, as the causative factor of the putative impairment of gluconeogenesis activity in *maf1Δ* has already been challenged [15,16,18]. The *maf1Δ* phenotype is reversed by a point mutation, resulting in the replacement of a glycine with alanine at position 1007 in C128, which is the second largest subunit of RNAP III [13]. The point mutation carried by *rpc128-1007* leads to a limited ability of this mutant to use glucose as a carbon source and significant induction of GCN4-regulated genes [15,16]. MAF1-deficient cells show a metabolic profile similar to some cancer cells which display the Warburg effect. Upregulated RNAP III activity causes cells to reprogram their metabolism towards increased activity of glycolytic enzymes when grown under high-glucose conditions, as shown by Szatkowska et al. [16]. The glucose flux is also redirected towards the pentose phosphate pathway, and the glycogen and trehalose shunt in *maf1∆* [16]. Conversely, in the *rpc128-1007* strain (with low RNAP III activity), the activities of all glycolytic enzymes are reduced [16]. This is correlated with a drastic inhibition of glucose metabolism and a change in the transcription profile of glucose transporters [15].

Similar to the *maf1∆* phenotype, null mutants of ADH1, SDH1, SDH2, SDH3, SDH4, and FUM1 genes do not grow on glycerol as a carbon source [19]. Therefore, being guided by our proteomics data, we aimed to decipher whether the tricarboxylic acid cycle (TCA) activity is impaired in MAF1-deficient cells cultured on glucose, on glycerol and after a temperature shift. This implication was additionally supported by the fact that the point mutation in the C128 subunit of RNAP III restores the ability of *maf1∆* to effectively utilize glycerol at 37 °C in the double-mutant *maf1∆ rpc128-1007*.

The main goal of this study was to assess the relative importance of the TCA cycle in *S. cerevisiae* strains that differ in the activity of RNAP III under fermentative and respiratory growth conditions and to explain the lack of *maf1∆* growth on respiratory carbon sources at non-permissive temperatures. Additionally, we aimed to understand the survival strategy of its suppressor, *rpc128-1007*, when the only available source of carbon is glucose. 

In this work we show that the metabolism of the *rpc128-1007* strain, decoupled from glucose signaling in the presence of a carbon source [15], relies on Idp-dependent reductive carboxylation of glutamine as the primary energy source, even though glucose is abundant. The low growth rate does not increase TCA cycle activity. Both mutants (*maf1∆* and *rpc128-1007*) show a decrease in TCA cycle activity during fermentative growth. Notably, both mutants accumulate acetyl-CoA. The observed changes in TCA cycle activity measured by the ^13^C flux approach and further supported with enzymatic assays are further reflected in the changes in NAD+/NADH ratios under both growth conditions studied here.

## 2. Results

### 2.1. ^13^C Flux through the TCA Cycle Is Affected Indirectly by RNAP III Activity on Glucose

To test the intracellular distribution of carbon flow through the TCA cycle in the *maf1Δ* and *rpc128-1007* yeast strains, the nuclear magnetic resonance (NMR) detected ^13^C isotopomer distribution in amino acids from ^13^C-labelling experiments was analyzed. For this purpose, different isotopic tracers, [U-^13^C] glycerol and [1,2-^13^C] glucose, were used.

The TCA cycle is a source of the carbon backbone for ten amino acids [20]. Consequently, the labelling distribution in amino acid pools depends on the ^13^C profile of TCA cycle intermediates. Alanine is used as a reporter molecule on isotopic labelling in pyruvate—the end metabolite of the glycolytic pathway. This approach was taken, owing to difficulties in measuring pyruvate levels by 2D-^1^H,^13^C HSQC NMR spectroscopy. When labelled alanine was observed, this enabled the carbon flux estimation before pyruvate entered the TCA cycle. The splitting of carbon labelling into alanine isotopomers gives information on the positioning of the isotopic atoms in the end-product of glycolysis. In the case of *rpc128-1007* a higher concentration of [3-^13^C] alanine could be observed, which means that this isotopomer might originate from the PPP pathway. 

While [1,2-^13^C] acetyl-CoA results in [4,5-^13^C] glutamate when generated directly from α-ketoglutarate via transamination, a full round of TCA cycle activity moves this label into [3-^13^C] and [1,2-^13^C]-labelled glutamate isotopomers. While glutamate is not a TCA cycle intermediate, it is a very good reporter of TCA cycle activity due to its high abundance. Specific isotopomers allow conclusions to be drawn on the relative activity of different pathways and flux distribution.

**Figure 1:**
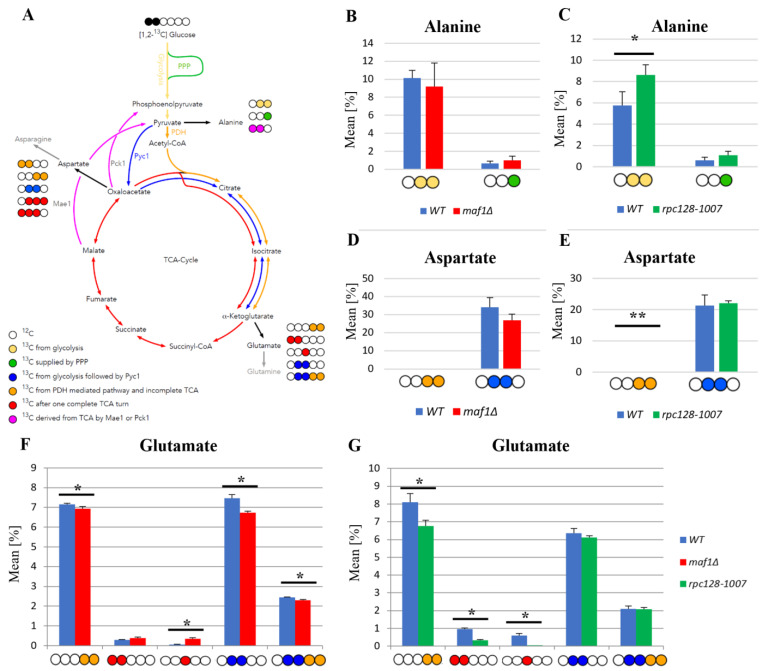
The MAF1 deletion and C128 point mutant yeast strains have a less active TCA cycle than WT during fermentative growth. (**A**) Schematic simplified diagram of the possible incorporation of ^13^C atoms from [1,2-^13^C] glucose to isotopomers of alanine and glutamate. Carbon atoms are shown as circles. Filled circles represent ^13^C incorporation into amino acid isotopomers, whereas empty circles represent ^12^C nuclei. Yellow circles stand for ^13^C derived from glycolysis. Green circles mark atoms supplied by the pentose phosphate pathway (PPP). Blue circles represent labelling derived from glycolytic pyruvate followed by the anaplerotic reaction mediated by pyruvate carboxylase 1 (Pyc1) into the TCA cycle. Amber circles show ^13^C obtained from acetyl-CoA formed from glycolytic pyruvate by the pyruvate dehydrogenase complex (PDH) and exiting the TCA cycle before completion of one full cycle. Red circles symbolize atoms that leave the Krebs cycle after at least one complete turn. Pink stands for labelling that exits the TCA cycle through cataplerotic pathways driven either by the malic enzyme (Mae1) or by phosphoenolpyruvate carboxykinase (Pck1) into pyruvate. Colored arrows symbolize the carbon flow that has led to specific labelling positions. Panels (**B**–**F**) show alanine (**B**,**C**), aspartate (**D**,**E**) and glutamate (**F**,**G**) ^13^C isotopomer percentage distributions in yeast strains with different RNAP III activity. Yeast cells were grown in rich medium (YP) supplemented with 2% [1,2-^13^C] glucose (YPD) until the log phase. Cultures were grown overnight in YPD (**B**,**D**) or YPGly (**C**,**E**) medium. Results are shown as the mean alanine, aspartate and glutamic acid isotopomer distributions with the standard deviation (SD) for three independent biological replicates. The unlabeled amino acid pools are not shown in the graph. An asterisk (*) indicates a *p*-value of <0.05, a double asterisk (**) marks *p*-values of ≤0.01 according to Student’s *t*-test.

Except for the outflow of intermediates to support the biosynthesis of glutamate, the synthesis of carbon flux into aspartate in *rpc128-1007* on glucose as well in *maf1Δ* is not greatly disturbed, as indicated by the relative percentage fold change of [3,4-^13^C] and [2,3-^13^C] aspartate (Figure 1D,E). No change in PDH- derived [3,4-^13^C] aspartate seems explicit, while the presence of [2,3-^13^C] aspartate, observed in *rpc128-1007*, suggests that aspartate is mostly synthesized here via an anaplerotic pathway (Figure 1E), via the oxaloacetate branch, formed directly from pyruvate by the pyruvate carboxylase (Pyc1). In the strain with compromised RNAP III activity (*rpc128-1007*), the carbon flow through Pyc1, supports the formation of asparatate, in contrast to *maf1Δ* (Figure 1D). In *rpc128-1007*, the Pyc1-mediated pathway is dominant while directing this carbon backbone towards sustained glutamate synthesis [2,3-^13^C] glutamate and [2,3,4,5-^13^C] glutamate (Figure 1G). 

To conclude, both the increase and the decrease in RNAP III capacity correlate with changes in TCA efficiency under fermentative conditions, which is a decrease in flux that supports our previously published data [16]. The flux into aspartate is sustained. Different dependencies can be observed under respiratory growth on glycerol as the carbon source, which is supported by data presented in the following sections. 

The flux distribution data (Figure 1) suggest that both strains with altered RNAP III activity face a reduction in TCA cycle activity in comparison to the wild-type isogenic strain when grown on glucose. The effect is rather mild in *maf1Δ*. This is clearly indicated by the labelling pattern of glutamine (Supplement Table S1), and especially by positional ^13^C depletion in [4,5-^13^C] and [2,3-^13^C] glutamate isotopomers in the glutamic acid labelled pool (Figure 1F,G) found in both mutants, showing replenished flux towards completion of the cycle. However, the increase in [3-^13^C] glutamate indicates the formation of glutamate after one complete TCA cycle in *maf1Δ*. This isotopomer is completely absent from NMR spectra in Figure 1G showing the flux distribution in *rpc128-1007*. Moreover, depletion, [1,2-^13^C], is observed in *rpc128-1007*. [3-^13^C] and [1,2-^13^C] glutamate isotopomers are synthesized when the carbon backbone is converted before α-ketoglutarate (AKG) leaves the citric acid cycle to be further converted into glutamate (Figure 1A); these are markers for sustained TCA cycle activity.

### 2.2. Lower Flux toward Glutamate in the MAF1-Deficient Mutant Can Be Compensated by Its Suppressor’s High Metabolic Activity in Glutamate Synthesis

**Figure 2:**
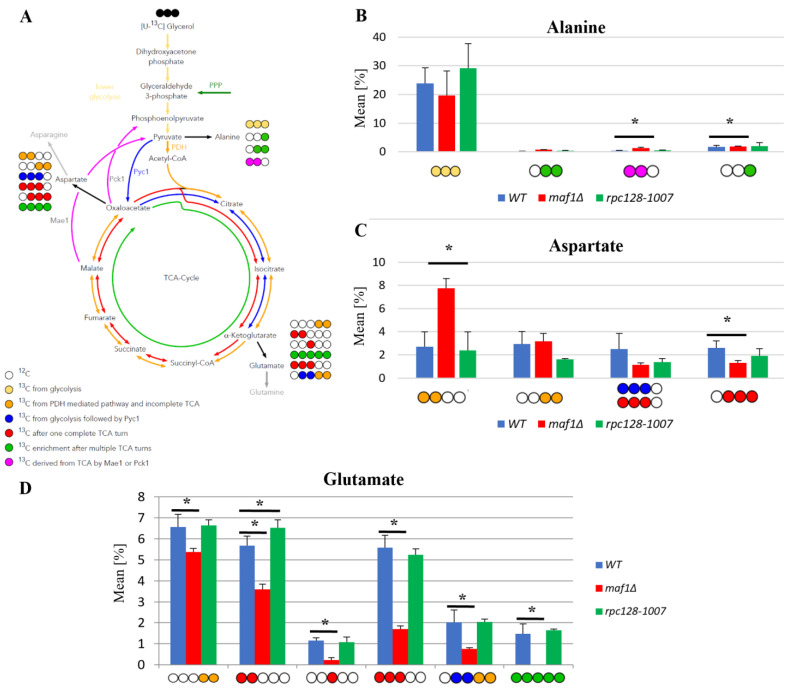
Higher TCA activity in *rpc128-1007* and lower in *maf1∆* compared to the reference strain on glycerol-based medium. Panels (**B**–**D**) show: alanine (**B**), aspartate (**C**) and glutamate (**D**) ^13^C isotopomer percentage distributions in yeast strains with different RNAP III activity. Yeast cells were grown in rich a medium (YP) supplemented with 2% [U-^13^C] glycerol (YPGly) (**B**–**D**) until the log phase. Cultures were grown overnight in YPGly (**B**–**D**) medium. Results are shown as the mean amino acid isotopomer distribution with the standard deviation (SD) for three biological replicates. The asterisk (*) indicates *p*-value of <0.05 according to Student’s *t*-test. The unlabeled glutamic acid pool is not shown in the graph. (**A**) Schematic simplified diagram of the possible incorporation of ^13^C atoms from [U-^13^C] glycerol into isotopomers of alanine, aspartate and glutamate. Carbon atoms are shown as circles. Filled circles represent ^13^C and empty circles represents ^12^C atoms. Blue circles represent labelling derived from glycolytic pyruvate followed by anaplerotic reaction mediated by Pyc1 into TCA. Amber circles show ^13^C obtained from acetyl-CoA formed from glycolytic pyruvate by PDH and exiting the TCA before completion of one full cycle. Red circles symbolize nuclei that leave the Krebs cycle after at least one complete turn. Green circles represent ^13^C enrichment that completed at least two full cycles of the TCA cycle before redirection of the carbon flow into biosynthesis pathways. Colored arrows symbolize the carbon flow that has led to specific labelling positions. An asterisk (*) indicates a *p*-value of ≤0.05 by Student’s *t*-test.

The ^13^C analysis clearly indicates that *maf1Δ* cells grown on glycerol at 30 °C have downregulated activity of the TCA cycle (Figure 2). This effect is reversed by its phenotypic suppressor, *rpc128-1007* via flux rewiring. The *rpc128-1007* shows highly active TCA on non-fermentable carbon source, which can be supported by an elevated biosynthesis of uniformly labelled glutamate (Figure 2D) that is formed after at least two full Krebs cycles. The data show that the [1,2,3-^13^C] glutamate pool is lowered by approximately 5 fold in *maf1Δ* and [U-^13^C] glutamate is not formed (Figure 2D). Moreover, in *maf1Δ* there is a significant decrease in both the [1,2-^13^C] and the [3-^13^C] glutamate pool, which are indicators of completion of at least one full TCA cycle. The glutamate concentration is still above the detection limit (Figure 2D). Additionally, the downshift in the [2,3,4-^13^C] aspartate isotopomer production indicates that the TCA has a lower capacity, and cannot be completed, in the MAF1-deficient strain (Figure 2C). However, it is worth noting that asparate production is sustained in this mutant, being synthesized along the route via PDH following the incomplete TCA cycle and evidenced by the [1,2-^13^C] aspartate isotopomer, which indicates the synthesis of metabolic precursors for nucleotide production (Figure 2C). The opposite activity of the TCA cycle is observed in the *maf1Δ* suppressor strain (*rpc128-1007*). This indicates an inverse correlation between RNAP III activity and citric acid cycle efficiency under respiratory growth. 

### 2.3. Elevated Temperature along with Non-Fermentative Glycerol Further Abolishes TCA Cycle Activity in maf1∆

We intended to use ^13^C flux analysis to precisely dissect metabolic alterations in *maf1∆* that cause the growth defect on glycerol at 37 °C. Therefore, cells carrying MAF1 deletion were grown on [U-^13^C] glycerol as a carbon source at 30 °C until OD600 ≈ 1.0 and the flux was also measured after 1 h shift to 37 °C, under the conditions which cause the mutant lethality.

**Figure 3:**
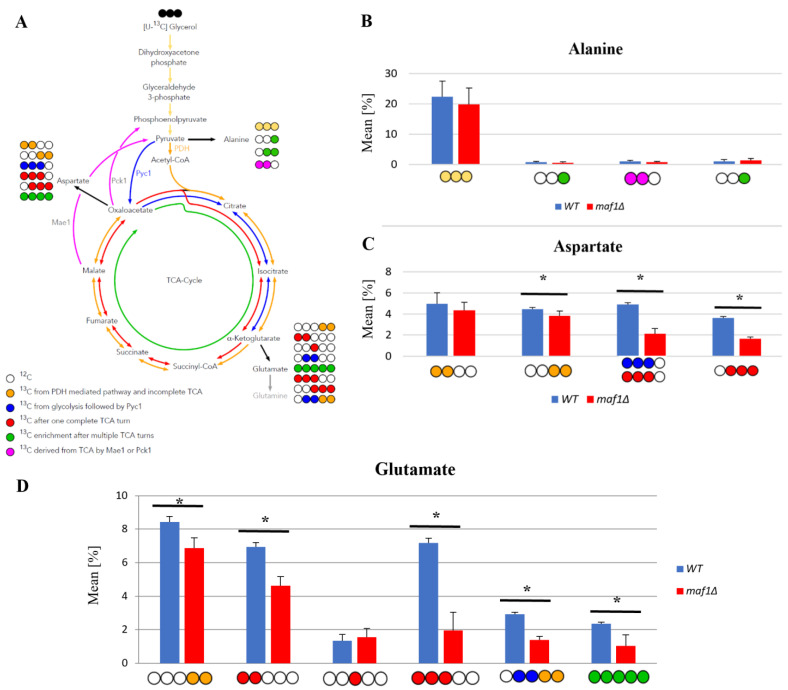
Lowered TCA activity under non-permissive conditions in the MAF1 knockout strain. Comparative analysis of ^13^C-labelling in alanine (**B**), aspartate (**C**) and glutamate (**D**) isotopomers. Yeast cells were grown in a rich medium (YP) supplemented with 2% [U-^13^C] glycerol (YPGly) (**B**–**D**) until the log phase. Additionally, cells were incubated for 1 h at 37 °C (**B**–**D**). Cultures were grown overnight in YPGly (**B**–**D**) medium. Data are shown as the mean percentage amino acid isotopomer distribution with the standard deviation (SD) from three independent biological replicates. As a simplification, the unlabeled amino acid fractions were omitted from the graph. (**A**) Schematic simplified diagram of the possible incorporation of ^13^C atoms from [U-^13^C] glycerol into isotopomers of alanine, aspartate and glutamate. Carbon atoms are shown as circles. Filled circles represent ^13^C and empty circles represent ^12^C atoms. Blue circles represent labelling derived from glycolytic pyruvate followed by cataplerotic reaction mediated by Pck1 into the TCA cycle. Amber circles show ^13^C obtained from acetyl-CoA formed from glycolytic pyruvate by pyruvate dehydrogenase complex (PDH) and exiting the TCA cycle before completion of one full cycle. Red circles symbolize nuclei that leave the Krebs cycle after at least one complete turn. Green circles represent ^13^C enrichment that completed at least two full cycles of the TCA cycle before redirection of the carbon flow into biosynthesis pathways. Colored arrows symbolize carbon flow that has led to specific labelling positions. An asterisk (*) indicates a *p*-value of ≤0.05 by Student’s *t*-test.

We noticed that in general the shift to the elevated temperature slightly upregulates the citric acid cycle activity in both the wild-type and *maf1Δ* yeast strains when compared to the data obtained at 30 °C (Figure 3B). The only exception was [1,2-^13^C] aspartate, which seemed to be enriched in *maf1Δ* grown at a permissive temperature (Figure 2C compared to Figure 3C). 

The most significant difference between *maf1Δ* metabolism at 30 °C and 37 °C, as we observed, was the consequent decrease in production of aspartate. *maf1Δ* grown on [U-^13^C] glycerol after the temperature shift produces less [1,2-^13^C], [3,4-^13^C], [2,3-^13^C] and [2,3,4-^13^C] aspartate isotopomers (Figure 3D) mirroring the deep dysfunction of the Krebs cycle. As indicated in Figure 3B, glutamate production was further adversely affected in all the cases of analyzed isotopomers visible in the NMR spectra. Given that amino acids such as glutamate and aspartate are particularly important for growth and proliferation, this level of downregulation of their synthesis might cause growth retardation but, in our opinion, shall not cause lethality. Thus, we presumed that MAF1-deficient cells redistribute carbon flux away from the TCA cycle at both permissive and elevated temperatures and we were interested in a detailed dissection of the flux rewiring.

### 2.4. Malic Enzyme Is the Key Enzyme Responsible for Cataplerosis in maf1∆ Affecting the Mutant Viability

Intriguingly, the high abundance of the PDH-derived [1,2-^13^C] aspartate isotopomer in *maf1∆* during oxidative metabolism was a reason to investigate the activity of TCA cycle enzymes. In this case, carbon flow through the Pck1-mediated pathway is an unlikely contributor, because [1,2-^13^C] phosphoenolpyruvate, formed from [1,2-^13^C] oxaloacetate, would drain away into gluconeogenesis. The PDH-based recycle hypothesis has also been indicated by our computer simulations. With computer simulations of a custom delta maf1FBA model, we were able to reproduce experimental observations and partially elucidate metabolic activity influenced by this important mutation. MAF1 knockout in silico was created by removing reactions that are activated by MAF1 [16,17] (Table S2). Computer simulations employed Constraint-Based Modelling in Surrey Flux Balance Analysis software (Surrey FBA Version 2.34) have showed increased flux via E1 alpha subunit Pda1 of the pyruvate dehydrogenase (PDH) complex, in the *maf1∆* strain model in silico (Table S3).

**Figure 4:**
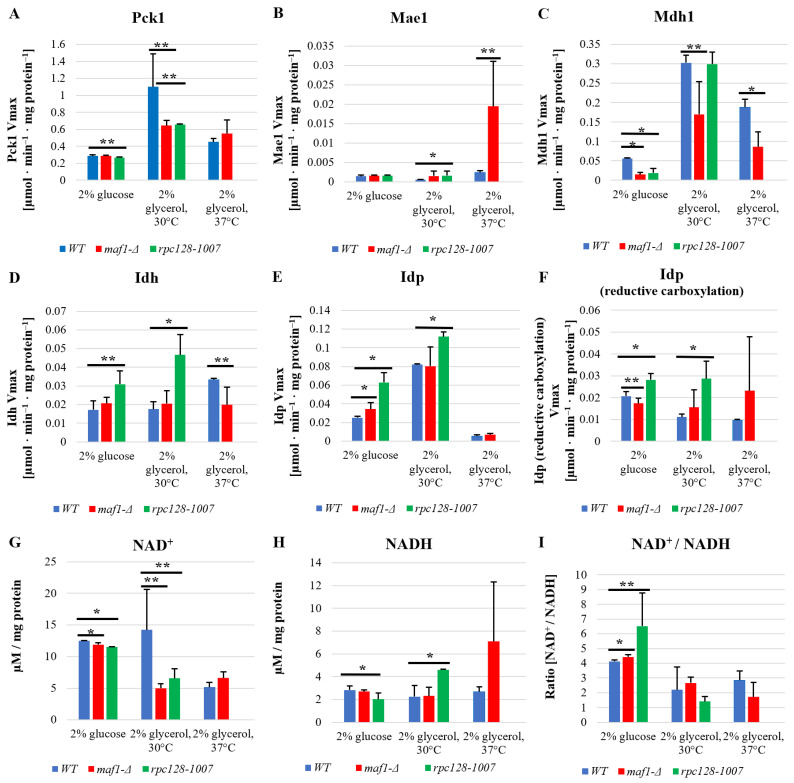
Under non-permissive conditions, Mae1 is a key enzyme responsible for cataplerosis in *maf1∆.* Idp-dependent reductive carboxylation of glutamine is proposed as an alternative metabolic strategy in *rpc128-1007* to maintain viability on the glucose medium. Yeast cells were grown in rich medium (YP) supplemented with either 2% glucose or 2% glycerol at 30 °C. To verify the phenotypic effect of MAF1 deletion, cells grown in YPGly were transferred for 2 h to 37 °C. All enzymatic activities were measured in cell-free yeast extracts (**A**–**F**). The reaction rates were monitored by measuring NADH concentration change over time at 340 nm. Protein concentration was assessed according to the Bradford assay. Data are expressed as the mean Vmax obtained from at least three independent biological replicates. NAD+ and NADH levels were measured according to NAD/NADH Assay Kit II (colorimetric, ab221821, Abcam) as stated in manufacturer’s protocol (**G**–**I**). NAD+ and NADH concentrations expressed in µM were calculated from standard curve and standardized to the total concentration of proteins expressed in mg. The + standard deviations (SD) are shown. Asterisks (*) indicate a *p* value of ≤0.05 and double asterisks (**) mark *p*-values of ≤0.01 by Student’s *t*-test.

In order to verify our hypothesis and further support our observations, we supplemented the ^13^C flux data with additional experimental data measuring the activity of several TCA cycle enzymes. Pck1 has been suggested to flux rewiring in the TCA cycle in *maf1∆* at non-permissive temperature [17]. However, as shown in Figure 4A, Pck1 enzymatic activity is decreased in *maf1∆,* and the temperature shift does not influence Pck1 activity in *maf1∆* in a non-fermentable carbon source. The direct cause of flux redirection away from gluconeogenesis seems to be the NADP+-dependent malic enzyme supported by malate dehydrogenase (Mdh1), since *maf1∆*, exhibits a reduction in Mdh1 activity regardless of the carbon source (Figure 4C).

A malic enzyme (Mae1) assay showed upregulated activity in *maf1∆* as well in *rpc128-1007* on non- fermentable carbon source both at 30 °C and 37 °C (Figure 4B). No significant changes were observed for Mae1 Vmax under fermentative conditions. Consequently, the results obtained do not fully support the hypothesis of Pck1 activity in TCA cycle flux redistribution, but may indeed serve as an additional constrain resulting in the observed flux redirection towards aspartate. *maf1∆* cells show an elevated carbon flux through a Mae1-mediated shunt producing pyruvate, which then enters the TCA cycle again, forming a futile cycle. 

Malate dehydrogenase (Mdh1) uses malate as a substrate and might compete for the substrate with Mae1. Mdh1 converts malate to oxaloacetate; however, when less active, this produces less substrate for Pck1-catalyzed reaction. 

According to our data, Mdh1 appears to be another key constrain in flux redirection towards pyruvate instead of phosphoenolpyruvate in the MAF1 gene-deleted strain, as we found that Mdh1 activity is significantly decreased in *maf1∆* when cells were grown in glycerol (Figure 4C). This result is consistent with the overall downregulation of the TCA cycle in the *maf1∆* mutant with high RNAP III activity. In contrast, a positive relationship can be seen between high Mdh1 activity and overall TCA cycle activity in *rpc128-1007* on the glycerol based medium, based on ^13^C flux analysis (Figure 4C).

### 2.5. Reductive Carboxylation of Glutamine Is the Survival Strategy of rpc128-1007 Grown on Glucose

Despite the decreased glycolytic activity the *rpc128-1007* strain is still able to grow on fermentable carbon source. [16]. This has been a puzzling observation to us requiring further investigation. In theory, when glycolysis is blocked, reductive carboxylation of glutamine may serve as an alternative source for metabolic intermediates [21] where citrate is formed from α-ketoglutarate (AKG) by isocitrate dehydrogenase (Idh or Idp). To determine whether reductive carboxylation of α-ketoglutarate to D-isocitrate is occurring in *rpc128-1007*, the enzymatic activity of NAD+-dependent isocitrate dehydrogenase Idh1-3 and NADP specific isocitrate dehydrogenase Idp1-3 was measured in both directions: the conversion of D-isocitrate to AKG, and of AKG to D-isocitrate (reductive carboxylation reaction).

We found that the activity of both Idh1-3 and Idp1-3 is upregulated in *rpc128-1007* regardless of the carbon source (Figure 4D). No activity in reductive carboxylation, which might be Idh1-3 dependent, was noted for any of the tested strains in our assaysAs is consistent with the working hypothesis, there is a significant increase in Idp1-3 activity in the reductive carboxylation direction in *rpc128-1007* regardless of the carbon source.

The intracellular redox potential is primarily determined by the NAD+/NADH ratio and to a lesser extent by the NADP+/NADPH ratio [22]. It has been shown by other authors that the reductive carboxylation of glutamine can be induced by an unbalanced cellular ratio of NAD+ to NADH [23]. Maintenance of the NAD+/NADH ratio is essential for mitochondrial function, therefore, we determined the ratio in the mutant strains. Firstly, as shown in Figure 4I, both strains with altered RNAP III activity have elevated NAD+/NADH ratios. An increased NAD+/NADH ratio in the cytoplasm has been reported as a hallmark of efficient glycolysis, which would be consistent with *maf1∆,* having high glycolytic activity [24]. In the case of *rpc128-1007*, which is unable to utilize glucose efficiently via glycolysis, the presented data strongly support the hypothesis of reductive carboxylation of glutamine as a metabolic survival strategy of *rpc128-1007*. On the other hand, under non-fermentable growth conditions, increased NADH levels are observed in *rpc128-1007*, supporting the view of increased efficiency of the TCA cycle in this strain as presented in Figure 4H. (the middle bars set). 

The NAD+/NADH ratio in MAF1-deficient cells seems to be perturbed under glycerol growth conditions at 37 °C. We presume that the activity of the PDH complex might contribute to the observed increase in NADH concentration in these mutant cells. 

### 2.6. maf1∆ and rpc128-1007 Show Enhanced acetyl-CoA and Lipids Content

Finally, to support the putative increase in the activity of PDH, we measured the level of cellular acetyl-CoA, as this metabolite is an intermediate link between glycolysis and the TCA cycle. Since measuring the concentration of acetyl-CoA in various cellular compartments, seems technically not trivial, many authors use the total level of this metabolite as an indicator of the nucleo-cytosolic pool of the compound in the cell [25,26]. 

To extract acetyl-CoA, the yeast cultures were grown in YPD or YPGly at 30 °C. Additionally, half of the cultures grown in YPGly were shifted from 30 °C to 37 °C for 2 h. The acetyl-CoA was extracted and measured according to the MAK039-1KT fluorometric kit (MilliporeSigma, St. Louis, MO, 63103, USA).

**Figure 5:**
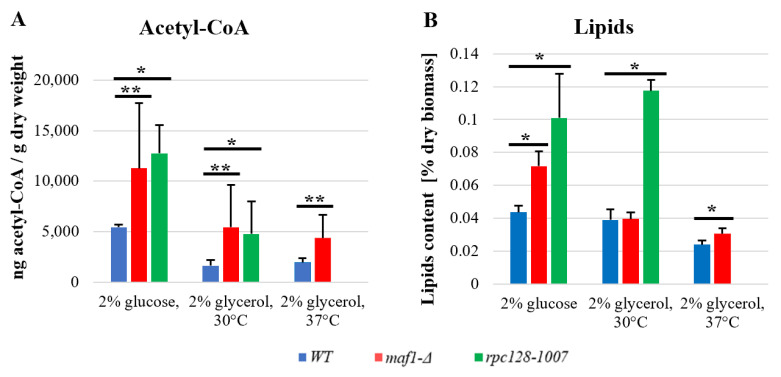
Acetyl-CoA concentration is higher in logarithmically growing yeast cells with altered RNAP III activity (**A**), which is consistent with lipids content on glucose (**B**). Yeast cultures were grown in YPD or YPGly medium until OD600 ≈ 1.0 at 30 °C. WT and *maf1Δ* cells that were grown in a glycerol-based medium until OD600 ≈ 1.0, were additionally transferred for 2 h to 37 °C. For measuring acetyl-CoA (**A**), the yeast cell metabolism was rapidly stopped by quenching in cold HPLC-grade methanol (−80 °C). Acetyl-CoA extraction was performed as was described in Materials and Methods. The concentration of acetyl-CoA was measured using an Acetyl-Coenzyme Assay Kit (MAK039-1KT, MilliporeSigma, St. Louis, MO, 63103, USA. The extraction of the total cellular lipids (**B**) was taken from the lyophilized biomass with chloroform: methanol mixture (2:1, *v*/*v*). Lipids were recovered by evaporation of the bottom layer solvent and pellets were weighted. The result obtained is the percentage of lipids content in the dry weight of the cells. All results are shown as the mean value from at least three independent biological replicates with the standard deviations (SD). An asterisk (*) indicates a *p*-value of ≤0.05 and a double asterisk (**) marks a *p*-value of ≤0.01 by Student’s *t*-test.

As indicated in Figure 5 the level of acetyl-CoA in yeast under glucose conditions is higher than on the non-fermentable carbon source, which was also reported earlier for wild-type cells [25]. Surprisingly, in both mutated strains, the acetyl CoA concentration is approximately 2-fold higher compared to WT (Figure 5A) when cells are in the log growth phase. The result obtained for *maf1∆* can be explained by increased activity of glycolysis [16], but the accumulation of acetyl-CoA in *rpc128-1007* seems unusual. Additionally, accumulation of acetyl-CoA in *maf1∆* can be supported by the deficiency of glyoxylate shunt revealed previously by proteomic data [16]; ordinarily, the glyoxylate shunt consumes acetyl-CoA to produce malate. The total cellular acetyl-CoA level measured in the strains with altered RNAP III under respiratory conditions is decreased (Figure 5A).

Acetyl-CoA is a precursor for synthesis of lipids. There are several studies from MAF1-deficient model organisms, including *C. elegans*, *S. cerevisiae* and human cells [27,28,29], which show that deletion of the MAF1 gene results in the accumulation of fatty acids or lipids. No published data exist so far indicating that this occurs in *maf1∆* cells or in cells with compromised RNAP III, which are capable of such accumulation when grown on non-fermentable carbon source. Therefore, the next step in our investigation was to determine the concentration of lipids in the mutated strains in glucose-based and glycerol-based media. 

The lipid content measured in *maf1∆* and *rpc128-1007* (Figure 5B) under fermentative growth conditions is corelated with an elevated concentration of acetyl-CoA in both strains, and it is increased compared to the control. A higher concentration of acetyl-CoA as well as of lipids, can also be observed in *rpc128-1007* grown under respiratory conditions and in *maf1∆* after a shift to 37 °C (Figure 5B). Despite the increased concentration of acetyl-CoA in *maf1∆* grown on the glycerol-based medium, the content of lipids per g of dry weight does not seem to be significantly higher. Lipid droplets are the major reservoir for lipids in *maf1∆* grown on glucose and on glycerol at a restrictive temperature. However, this does not seem to be the case for *rpc128-1007* as indicated by microscopic investigation, which might be a manifestation of significant changes in the lipid composition of cell membranes (Figure S1).

## 3. Discussion

Mitochondrial dysfunction has been associated with major civilization diseases including type 2 diabetes, Alzheimer’s disease and several other neurodegenerative disorders and, therefore, accelerated health problems in old age [20,30]. The molecular basis of disease is different and involves different levels of regulation of cellular activity. Due to systems biology approaches applied in basic research, we are closer than ever to answering the fundamental question of how metabolic incapability is correlated with non-coding gene regulation, in order to understand the health or disease-related physiology at a cellular level. 

In the case of the RNAP III-compromised strain (*rpc128-1007*) with a phenotypic growth defect on glucose, we previously successfully explained its growth defect on glucose at the level of enzyme activities and enzyme abundance [16]. We also established a new metabolic link between mitochondrial proteome abundance and RNAP III-driven non-coding RNA synthesis. Further under the course of this study, we intended to characterize mitochondrial activity in depth to address several urgent questions related to the phenotypic dependence on carbon source and mortality at higher temperature.

The focus of our current report was to investigate the possible impact that RNAP III activity has on mitochondrial metabolism and carbon flux distribution in the TCA cycle under different growth conditions. Our previously reported observations related to the mitochondrial proteome in the mutant strains with non-optimal RNAP III activity (*rpc128-1007* and *maf1∆)* highlighted a possible scenario for alterations in the mitochondrial metabolism that required further investigation at metabolic level to enable a conclusion to be drawn on its activity. 

In this study, we show that RNA polymerase III overactivity as well as its downregulation can adversely affect TCA cycle flux via TCA cycle enzyme activities in *S. cerevisiae* when RNAP III activity is not optimal for appropriate growth conditions. Compromised RNAPIII activity seems to lower mitochondrial TCA cycle activity when compared to wild-type cells, if glucose is abundant. However, the C128 mutation favours growth on non-fermentable carbon sources. 

We recognize the potential of the precise optimization of RNAP III activity towards balancing aberrant mitochondrial metabolism by the manipulation of the holoenzyme activity, but not by its full inhibition. In our opinion, non-optimal RNA production driven by RNAP III is a superior signal compared with the extracellular glucose signaling in *S. cerevisiae*, an observation we reported previously [15]. However, the idea seems very ambitions, if we take into account that the activity of RNAP III in *S.cerevisiae* is regulated also on a chromatin level by recently discovered chromatin-associated regulator Fpt1 and that the activity is also highly dynamic and tissue-dependent in metazoans [31].

The reprogrammed ncRNA transcription is followed by proteome reprograming, highlighting a putative role of tRNA/5S RNA in the observed changes in *rpc128-1007* and *maf1∆* via the translation process [16]. Firstly, translation is tightly bound to nutrient availability via tRNA post-transcriptional metabolism, the conserved chemical modifications [32], secondly tRNA modifications impact tRNA cleavage [33]. Several tRNA fragments (tRFs) have been detected in yeast under oxidative stress, methionine starvation, nitrogen starvation, heat shock, and entry into the stationary phase [34,35]. The interaction of 5′-tRFs and 3′-tRFs with ribosome-associated aa-RSs correlate with impaired efficiency of tRNA aminoacylation [36]. Hypothetically, tRFs and the other small non-coding transcripts of RNAP III, can interfere not only with translation apparatus, but also with RNA-binding enzymes of metabolic pathways and can modulate their activity in *S.cerevisiae*. [37]. The glycolytic enzymes and TCA cycle enzymes bind nuclei acids. Ryazanov, Entelis et al. and Asencio et al. among many others, have shown that RNA including tRNA, interact with glyceraldehyde-3-phosphate dehydrogenase (TDH/GAPDH) as well as with enolase, with both enzymes affecting mitochondrial metabolism [37,38,39]. Recently made available data on small non-coding interactome, includes most of the glycolytic enzymes from yeast and TCA cycle enzymes such as ACO1, IDP1, LPD1, LSC1 and finally MDH1, the enzymatic activity of which we found being adversely affected in *maf1∆* [37].

Most of the RNAP III products, expressed in bulk, are the tRNAs (275 actively transcribed genes) and 5S rRNAs; therefore, it is very likely that non-optimal RNA synthesis of this class of transcripts, in tandem with post-transcriptional processing, is likely to have a significant global effect on the cell’s metabolism, followed, as we observe, by systemic metabolic adjustment of mutant cells, which causes the cells to lack plasticity at adopting either fermentable or non-fermentable carbon.

Among others, the RNAP III-synthesized transcripts contributing to RNA processing in yeast are RNase P RNA and snoRNA, whereas RNase MRP RNA processes mitochondrial pre-rRNA in mammals. 

### 3.1. RNAP III Non-Optimal Activity Affects the TCA Flux and TCA Enzymes Activities

**Figure 6:**
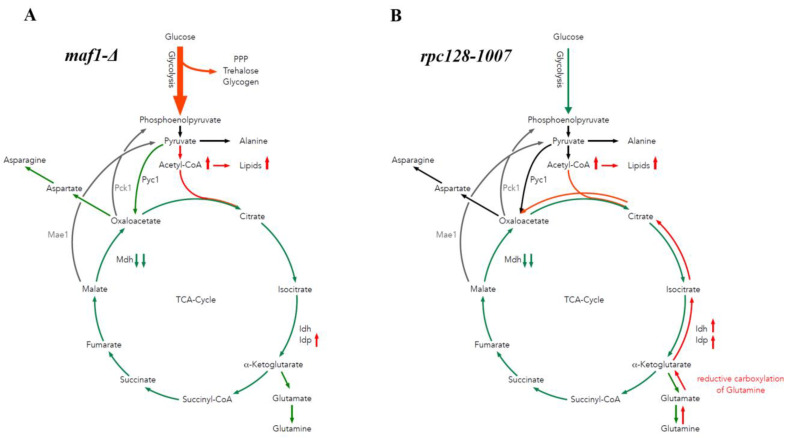
Model of carbon flow through the TCA cycle in MAF1 deletion and *rpc128-1007* mutants grown on glucose. During fermentative growth on glucose, the MAF1 knockout strain exhibits accelerated glycolysis (**A**) compared to the WT [16]. Glucose flux is redirected into PPP, trehalose, glycogen, and glycerol biosynthesis [16] and acetyl-CoA and lipid formation (Figure 5). TCA cycle activity is slightly diminished in the MAF1 knockout strain due to carbon being redirected towards glutamate biosynthesis (Figure 1F). While growing on glucose, the *rpc128-1007* (**B**) mutant shows downregulated glycolytic activity [16] in comparison to the reference isogenic strain. Additionally, the citric acid cycle activity is lowered (Figure 1G). TCA cycle activity is supported by the Pyc1-mediated pathway and Idp-dependent reductive carboxylation of glutamine, which results in an elevated acetyl-CoA pool to support lipid biosynthesis. Green represents a diminished carbon flux and red represents an elevated flux compared to the WT. Abbreviations: TCA—citric acid cycle Pck1—phosphoenolpyruvate carboxykinase 1, Mae1—malic enzyme 1, Mdh—malate dehydrogenase, Pyc1-pyruvate carboxylase, Idh—NAD+-specific isocitrate dehydrogenase, and Idp—NADP-specific isocitrate dehydrogenase.

It is believed that yeast cells lower the level of TCA cycle activity on glucose, regardless of oxygen availability [40,41]. The generally held view of a catabolite-repressed TCA cycle in glucose-excess cultures of *S. cerevisiae* contrasts with Blank and Sauer’s observation that although the TCA cycle genes are subject to glucose repression [42] the relative respiratory activity of the TCA cycle may increase even at a high glucose concentration provided the growth rate or the glucose uptake is impaired [43]. Our data support the view represented by Blank and Sauer, claiming that the regulation of TCA cycle activity is not under transcriptional control of the genes that are subject to glucose repression. In contrast to Blank and Sauer’s observations on TCA cycle activity in CEN.PK113-7D genetic background, we do not observe increased TCA cycle activity in the *rpc128-1007* strain with the compromised RNAP III activity (Figure 6B). Cells with compromised RNAP III (*rpc128-1007*) grown in glucose, show increased transcription of high-affinity glucose transporters HXTs and reduce concomitantly their growth and uptake of glucose with no increase in TCA cycle activity (Figure 1G) [15,16]. This suggests that other factors than growth rate or glucose uptake determine the TCA cycle activity in *S. cerevisiae*, undoubtedly an intracellular factor.

The broad change in the abundance profile of enzymes engaged in the TCA cycle seems to affect *maf1Δ* TCA cycle activity rather less when compared to its suppressor (*rpc128-1007*) (this study). This suggests that the control of TCA cycle activity is not per se enzyme abundance dependent. Other factors (proteins localization, metabolites, non-coding tRNAs) may be in place [44]. In yeast strains with altered RNAP III activity (*maf1Δ* and *rpc128-1007*), the carbon flux through the citric acid cycle is diminished more than in the reference strain under high-glucose conditions (Figure 6A,B). The low percentages participation of [2,3-^13^C] aspartate, [1,2-^13^C], [2,3-^13^C], [3-^13^C], [4,5-^13^C], [2,3,4,5-^13^C], -glutamate isotopomers (Figure 1E–G, Table S1), suggests that the carbon flux through the TCA cycle is not only directed towards biosynthesis of glutamate, but also towards different metabolic routes unlike in the wild-type strain. The change in TCA cycle activity is reflected by flux direction. Glutamate is synthesized in cells with compromised RNAPIII (*rcp128-1007*) via the Pyc1 shunt. In the MAF1-deficient mutant, isotopomers of glutamate are more efficiently formed via the PDH-mediated pathway (Figure 1G,F and Figure 6B). 

Additionally, a higher concentration of the nucleo-cytosolic pool of acetyl-CoA was also confirmed for *maf1∆* (Figure 5A). The distribution of pyruvate-derived alanine isotopomers in *maf1∆* does not show an increased concentration of the end-point glycolytic metabolite, pyruvate. However, in addition to alanine biosynthesis, pyruvate can be converted into ethanol or acetyl-CoA. No increase in ethanol synthesis was observed in *maf1∆* [16]. Therefore, the high levels of acetyl-CoA (Figure 5A) found in *maf1∆* point at pyruvate, as an abundant metabolic source channeled into acetyl-CoA. The increase in acetyl-CoA concentration guides cellular metabolism towards biosynthesis processes, e.g., by transcriptional mechanism, and shuts off catabolic reactions [45]. This effect of redirecting metabolism towards anabolic reactions is supported by the observation of lipids accumulation in lipid droplets (Figure S1). According to several studies on model organisms including *C. elegans*, mammalian cells and *S. cerevisiae* [27,28,29,46], MAF1 negatively regulates lipid metabolism. Consequently, we assume that the increased accumulation of acetyl-CoA and lipids in *maf1∆* is a result of increased glucose uptake and increased activity of the PDH complex, as suggested by the modelling. 

Despite low glycolytic activity observed in *rpc128-1007*, high concentrations of acetyl-CoA and accumulated lipids (Figure 5B) can also be observed in *rpc128-1007*. The collected data imply that reductive carboxylation is the potential source of cytosolic acetyl-CoA in *rpc128-1007* (Figure 6B). We confirmed that the enzymatic activity of both isocitrate dehydrogenase, NAD+-specific Idh and NADP-specific Idp, is upregulated in the mutant strain grown on glucose (Figure 4D,E). Idp, but not Idh, also has increased activity in the reductive carboxylation reaction (Figure 4F). Therefore, we favor the scenario that reductive carboxylation of glutamine in *rpc128-1007* depends on Idp cellular activity. 

Reductive carboxylation of glutamine is an alternative metabolic pathway, where citrate synthesis occurs by a reductive carboxylation of α-ketoglutarate (AKG) catalyzed by isocitrate dehydrogenase (Idh or Idp) at the expense of citrate formation in the pyruvate decarboxylation reaction. Cells utilize glutamine to sustain TCA cycle cataplerosis, biosynthesis of nucleotides and fatty acids and to maintain the cellular redox balance [47]. Glutamine is utilized at a high rate by rapidly growing cells. For instance, glioblastoma cells have upregulated aerobic glycolysis as well as an active TCA cycle with a strong carbon efflux into fatty acid biosynthesis pathways. These cells exhibit a very high catabolism of glutamine, which is a source of the carbon backbone for TCA cycle replenishment. Therefore, citrate generated by reductive carboxylation may be used for lipid biosynthesis in the cell [48]. The reductive metabolism of glutamine or glutamate for lipid biosynthesis allows cells to save the glucose-derived carbon for the production of biosynthetic precursors such as ribose, which is not usually synthesized via other routes [49]. Glutamine is a carbon backbone source for the biosynthesis of lipids and acetyl-CoA was previously reported for a brown adipocyte cell line [50] and A549 cells growing under hypoxia [49].

The concentration of NAD+ and NADH measured in *rpc128-1007* grown on glucose is decreased compared to the wild type. Despite this, we see a significantly increased NAD+/NADH ratio in this mutant strain (Figure 4I). In our opinion, the change in the NAD+/NADH ratio in the cell supports lipid biosynthesis possibly because of impaired utilization of NADH by the mitochondrial respiratory chain induced by the reductive glutamine carboxylation [23]. This redox imbalance further suggests that the mitochondrial respiratory chain might be dysfunctional in the *rpc128-1007* strain grown on glucose. 

Here, we depict first that accumulation of lipids can be observed in the *rpc128-1007* strain in a high-glucose medium. Therefore, we presume that the synthesis of different classes of RNA and their abundance might be an intracellular signal stimulating lipid accumulation of different classes of lipids, for instance, lipids building the *S. cerevisiae* membranes, not necessarily involving the formation of lipid droplets. 

It is likely that changes in the content of lipids in yeast strains with different tRNA levels are connected to tRNA metabolism or lipids modification. With the exception, of the role of aminoacyl transfer RNAs (aa tRNA) in translation, the transfer RNAs in bacteria were shown to participate in membrane lipid aminoacylation [51]. An in vitro study on tRNAs has shown an association of yeast tRNA with phospholipid bilayers [52]. More recent research has shown that lipids can protect RNA and modulate ribozyme activity via RNA–lipid interactions [53]. 

To conclude, the perturbed activity of RNAP III indirectly reduces TCA cycle activity during fermentative growth. Both mutant strains accumulate lipids and its precursor acetyl-CoA. By contrast, different routes lead to the observed increase in lipid accumulation, in *maf1∆* in *rpc128-1007* (Figure 6A,B).

### 3.2. The Difference in Growth of the Mutants with Altered RNAP III Activity on a Non-Fermentable Carbon Source Results from Flux Rerouting

As indicated by our study, the metabolic strategy that helps yeast cells with compromised RNAP III activity (*rpc128-1007*) metabolize glycerol efficiently and partially overcome growth retardation on glucose, is primarily flux redirection. Therefore, the mutant deprived from MAF1 when crossed with *rpc128-1007* can adjust its growth towards a more efficient metabolism of a non-fermentable type of carbon source. 

**Figure 7:**
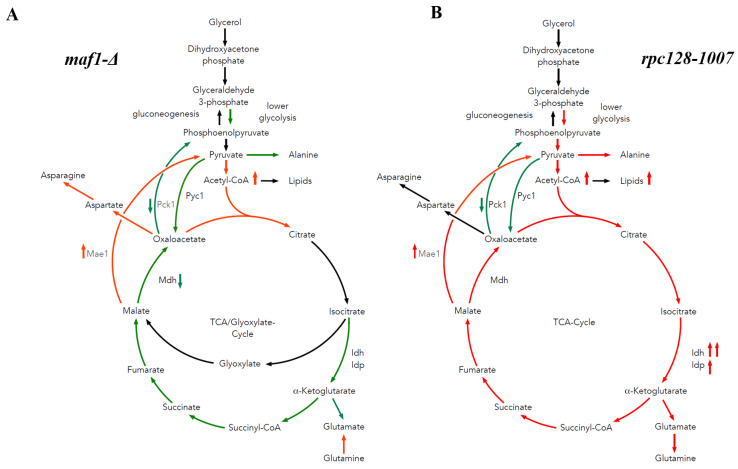
Proposed model of carbon flow through the TCA cycle in MAF1 deletion and *rpc128-1007* mutants grown on glycerol. On the non-fermentable carbon source, yeast strains with changed RNAP III exhibit contrary metabolic adjustments. The MAF1 deletion strain shows diminished activity in lower glycolysis, and the TCA cycle including anaplerotic pathways (**A**). The TCA cycle is additionally replenished by a highly active Mae1-mediated pathway. Acetyl-CoA levels are higher (Figure 5A) than in the wild type (**A**), which does not correlate with lipid levels. Meanwhile, *rpc128-1007* has highly active lower glycolysis and TCA cycle (**B**), including acetyl-CoA and lipid accumulation (Figure 5B) and reaction directed by PDH. Yet, the Pyc1-mediated anaplerotic pathway seem to be less active than in the reference strain. Malate is directed towards pyruvate by the Mae1-mediated reaction, rather than aspartate biosynthesis pathway (**B**). Green represents diminished carbon flux, while red stands for elevated flux when compared to WT. Abbreviations: TCA—citric acid cycle, Pck1—phosphoenolpyruvate carboxykinase 1, Pyc1- pyruvate carboxylase, Mae1—malic enzyme 1, Mdh—malate dehydrogenase, Idh—NAD+-specific isocitrate dehydrogenase, and Idp—NADP-specific isocitrate dehydrogenase.

As hypothesized, the study of *maf1Δ* and its suppressor by ^13^C flux analysis in the presence of uniformly labelled glycerol, showed that, unlike *maf1Δ*, its suppressor displayed higher TCA cycle activity (Figure 2A and Figure 7A,B). In *maf1Δ*, downregulation of the complete TCA cycle is observed deducted from the splitting of ^13^C NMR signals in the percentage fraction of [2,3,4-^13^C] aspartate derived from one complete TCA cycle, and the formation of [1,2-^13^C] aspartate and [3,4-^13^C] aspartate as a result of incomplete TCA cycle (Figure 2C). The increase in the [1,2-^13^C] aspartate pool concomitantly observed with the highly pronounced reduction of flux towards glutamate biosynthesis in response to the glycerol-based medium (Figure 2D) suggests that the TCA cycle operates as a bifurcated pathway to satisfy the requirement for biomass precursors (aspartate). In this case, in *maf1Δ*, the TCA cycle would not operate as a cycle, but rather as a two-branched pathway, similar to batch-growing cells, and in contrast to chemostat conditions [54]. The synthesis of aspartate can be additionally supported by glutaminolysis, which takes place in all proliferating cells [55].

The low percentages participation of [3,4-^13^C], [2,3,4-^13^C] and [1,2,3-^13^C]-aspartate as well as all the detected glutamate isotopomers in *maf1Δ* after a temperature shift (Figure 3C,D, Table S1), suggests that the carbon pool in the TCA cycle is directed not only towards biosynthesis of glutamate but also aspartate unlike in the *maf1Δ* at a permissive temperature.

On a glycerol-based medium, both the *maf1∆* and *rpc128-1007* strains show increased levels of acetyl-CoA (Figure 5A). Since acetyl-CoA activates Pyc1, the reduced carbon flux through the Pyc1-driven pathway can be explained either by a high amino acid abundance provided by the TCA cycle (the most probable for *rpc128-1007*) or by a higher pyruvate pool (suggested for *maf1∆*).

**Figure 8:**
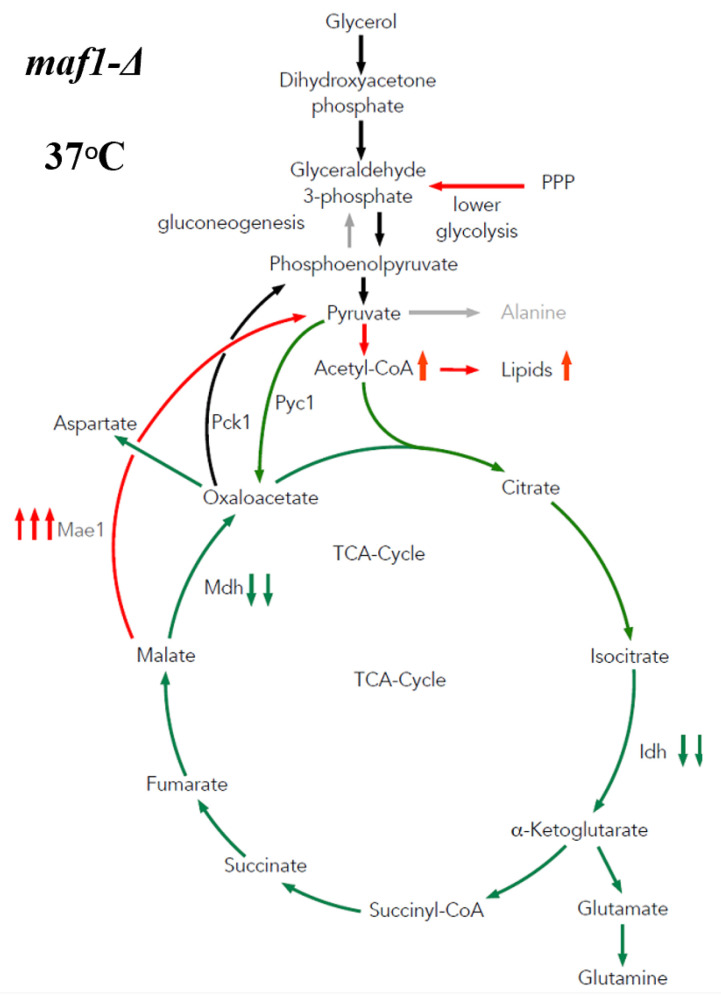
Proposed model of carbon flux through the TCA cycle during growth arrest of *maf1∆* cells under non-permissive conditions. Gluconeogenetic flux is redirected into the Mae1-catalyzed pathway towards pyruvate acetyl-CoA and lipid biosynthesis. Downregulation of Mdh1 is followed by a decrease in the conversion of malate into oxaloacetate. The green color represents lowered carbon flux, while red stands for enhanced activity of the pathway. Abbreviations: TCA—citric acid cycle, Pck1—phosphoenolpyruvate carboxykinase 1, Pyc1- pyruvate carboxylase, Mae1—malic enzyme 1, Mdh—malate dehydrogenase, and Idh—NAD+-specific isocitrate dehydrogenase.

Under respiratory conditions, the pyruvate fraction can be supplied by cataplerotic pathways involving activity of Pck1 and Mae1. We show here that Pck1 activity is lowered in both *maf1∆* and in its suppressor strain *rpc128-1007*, while Mae1 activity is higher in both mutants at high non-fermentable carbon source concentrations (Figure 4B). Furthermore, the downregulation of Mhd1 activity (Figure 4C) and the significant increase in Mae1 in *maf1∆* on glycerol after the shift to 37 °C contributes to flux directing towards pyruvate and fueling acetyl-CoA biosynthesis (Figure 8). We demonstrate increased lipid biosynthesis in the *maf1∆* strain, after a temperature shift of glycerol growing *maf1∆* culture to 37 °C (Figure S1). 

Mae1 is a major participant in lipid biosynthesis and it contributes to the development of obesity and type 2 diabetes [56]. It contributes to the NADPH pool and is engaged in buffering of ROS via NADPH-dependent recycling of the glutathione/thioredoxin pathways, working together with other NADP-recycling cytosolic enzymes such as G6PD, 6PGD, IDH1 and MTHFD [57,58]. The biosynthesis of membrane components such as fatty acids and cholesterol and the pyruvate–malate cycle provide essential support for cancer cell proliferation and migration in Mae1-dependent cancer cell metabolism [59]. It is worth investigating under which circumstances a significant increase in activity of Mae1, a pro-oncogenic enzyme in various tissues, accompanied by inhibition of Mdh in a proliferative cell line can cause lethality. 

Analysis of the liver of MAF1 knockout mice (which is metabolically similar to yeast cells grown on non-fermentable carbon source) by metabolomic profiling showed that most of the glycolytic and TCA cycle intermediates were not affected by the deletion, with the exception of pyruvate and acetyl-CoA, where levels were diminished [2]. This is contrary to the observations in *maf1∆* cells presented in this work (Figure 5A). Contradictory to findings presented here, in the KO mice liver, enhanced carbon flux through the TCA cycle was observed [2]. This may indicate that although the alteration in RNAP III activity affects carbon metabolism in eukaryotes, the endpoint metabolic effects may be species dependent. However, MAF1 activity in *C. elegans* leads to lipid accumulation similar to *maf1∆* cells [27]. Mammalian MAF1 has extended roles in higher eukaryotes, regulating expression of the non-coding vault tRNA, Y RNA, and 7SK genes, whose transcripts are not present in single-cell systems. MAF1 is recruited to RNAP II-dependent promoters as well and genes regulated by MAF1 additionally undergo tissue-specific expression such as the tDNA genes. In contrast to the contradictory observation in acetyl-CoA levels in the MAF1-deficient models (the single-cell yeast and vertebrate), the KO mice metabolism shows similarity to the *rpc128-1007* metabolism; therefore, an alternative hypothesis exists that KO mice might have acquired compensating mutations.

Since our data from *S. cerevisiae* cells partially contest the published data on mitochondrial metabolism in KO mice maf1+/−, we think it is worth reinspecting the metabolism using other, well-studied eukaryotic model organisms to answer the question of whether a lack of MAF1 protein correlates with dysfunction of mitochondrial metabolism and obesity or tumorigenesis.

## 4. Materials and Methods

### 4.1. Yeast Culturing and Metabolites Extraction for ^13^C Flux Analysis by Nuclear Magnetic Resonance (NMR) Spectroscopy

A twice-concentrated rich (2 × YP) medium without carbon source (pH 5.0) was prepared. In addition, 100 mM potassium hydrogen phthalate (C8H5KO4) was added to the medium to compensate pH fluctuations of the medium [60]. Prior culturing yeast strains [1,2-^13^C]-glucose (453188, Sigma-Aldrich, St. Louis, MO, 63103, USA) and uniformly labelled [U-^13^C]-glycerol (489476, Sigma-Aldrich, St. Louis, MO, 63103, USA) were suspended in Milli-Q water to a final concentration of 4% and sterilized by filtration. The 2 × YP medium was mixed with glucose or glycerol solution (1:1) to obtain 2% final concentration of ^13^C-labelled carbon source in the medium.

Overnight yeast cultures were centrifuged and washed twice with the YP medium without the carbon source. Yeast cells grown overnight at 30 °C (250 rpm) in YPGly were transferred to YPGly medium with [U-^13^C]-glycerol. For determining the carbon flux on glucose, yeast cells were grown overnight in YPD. To avoid revertants, the *rpc128-1007* and reference strains were grown overnight in YPGly. Next, the *rpc128-1007* and WT strains were shifted to YP with [1,2-^13^C]-labelled glucose. Yeast cultures with the ^13^C-labelled carbon source were inoculated to a maximum of OD600 ≈ 0.03 [61] and grown until OD600 ≈ 1.0 at 30 °C, 250 rpm. Half the volume of the WT and *maf1Δ* cultures grown on the non-fermentable carbon source were shifted for 1 h to 37 °C in order to verify the metabolic effect of MAF1 deletion. A volume of 5 mL of culture was rapidly quenched in 25 mL HPLC-grade methanol (−80 °C, 34860, Sigma-Aldrich, St. Louis, MO, 63103, USA) and centrifuged for 10 min (10,000 rpm, −20 °C). Pellets were washed with cold (−80 °C) methanol, frozen in liquid nitrogen (−196 °C) and stored at −80 °C until extraction. 

For metabolite extraction, the pellets were resuspended in 500 μL of a 1:1 mixture of HPLC-grade methanol (−80 °C) and water (4 °C) and homogenized with a Mini-Beadbeater 24 (Biospec products) using 200 μL of glass beads (425–600 μm; G8772, Sigma-Aldrich, St. Louis, MO, 63103, USA) in 8 bursts of 10 s each with breaks between the cycles for 1 min incubation on ice. Next, 500 μL chloroform (−20 °C) was added. Extracts were vortexed for 2 min (4 °C) and centrifuged for 30 min, 13,000 rpm (4 °C). The upper, polar phase was transferred into 2 mL tubes. The samples were frozen in liquid nitrogen (−196 °C) and freeze-dried in Alpha 1–2 LD lyophilizator (Christ, Chair of Analytical Chemistry).

### 4.2. NMR Spectroscopy

The extracted and dried samples were dissolved in 120 μL of NMR buffer (100 mM sodium phosphate buffer pH 7.0, 0.2 mM SDS, 8 mM imidazole, 0.03% sodium azide and 7.14% deuterium water D2O). A volume of 35 μL of samples were then transferred into 1.7 mm NMR tubes using a Gilson robot. Samples were sonicated for 10 min. 

All NMR spectra were acquired on an Avance III Bruker 600 MHz (14.1 T) NMR spectrometer equipped with a 1.7 mm TCI cryoprobe. Spectra were acquired at a temperature of 300 K. Samples were stored at 6 °C in a SampleJet automatic sample changer prior to data acquisition. 1D-^1^H NMR spectra were acquired using a 1D-NOESY pre-saturation pulse sequence. A total of 16,384 complex data points was acquired, the spectral width was set to 12 ppm, and the interscan relaxation delay was set to 4 s. A total of 16 steady-state scans and 128 transients were acquired. Prior to the Fourier transform, all free induction decays (FIDs) were apodised using an exponential window function with a line-broadening of 0.3 Hz, before FIDs were zero-filled to 131,072 data points. After the Fourier transform, all 1D-1H NMR spectra were manually phase corrected.

Ultra-high-resolution 2D-^1^H,^13^C HSQC NMR spectra were acquired using a gradient-selected 2D-^1^H,^13^C HSQC NMR pulse sequence using the echo/anti-echo scheme for quadrature detection. A total of 8 steady-state scans and 2 transients were acquired. A total of 30% of 8192 complex increments were acquired (2548 complex increments) using a non-uniform sampling scheme. The spectral widths were set to 13 ppm for the ^1^H dimension and 160 ppm for the ^13^C dimension, the interscan relaxation delay was set to 1.5 s. 

The 2D-^1^H,^13^C HSQC NMR spectra were reconstructed using the IRLS algorithm with 20 iterations with MDDNMR (version 2.5) [62,63] and then processed using NMRPipe (Version 9.2) [64]. Analysis of the signal multiplets in the 2D-^1^H,^13^C HSQC NMR spectra included quantum mechanical simulation of the NMR multiplets and isotopomer analysis and was performed by the MetaboLab software (Version 2023.0529) [65]. Based on ^13^C-^13^C J-coupling patterns, ^13^C isotopomer distributions were calculated based on the approach described by Chong et al. [66].

### 4.3. Metabolism Modelling 

A total of 12 genome-scale models of the *S. cerevisiae* metabolic network published since 2003 according to B. Heavner, 2015 [67] have been compared. The version chosen was a consensus yeast model Yeast7.6. A perl script allowed us to transfer annotations from the yeast 7.0 model to the yeast 7.6 model to be used in the SurreyFBA environment [68]. A simple procedure for locating genes was established: (1) Take the gene name from YDB, (2) find it in the “Reaction” tab in the “Rule” column, and (3) write the reaction Ids. The Delta_maf1FBA model was designed by removing reactions that are activated by MAF1. These reactions are indispensable for gluconeogenesis and amino acids biosynthesis pathways. *maf1Δ* leads to a decrease in mRNA levels of FBP1, HXT6&7, LYS9, MET2, PCK1 and accumulates glycogen and trehalose, and enhances production of glycerol [16,17]. FBA and FVA analyses were performed. 

### 4.4. NADH-Linked Enzymatic Assays

Yeast cultures and cell-free extracts were prepared as previously described [16,69]. Reaction mixtures and reagents, used to start the reaction, were different for each enzyme and had the composition as described below.

NAD+-dependent isocitrate dehydrogenase (Idh1, Idh2, Idh3, EC 1.1.1.41) activity was measured in cell-free extracts according to protocol described by Lin and co-authors [70] The reaction towards α-ketoglutarate (AKG) contained 40 mM Tris-HCl pH 7.4, 0.5 mM NAD+ (N7004, MilliporeSigma, St. Louis, MO, 63103, USA), and 4 mM MgCl_2_. The reaction was started with the addition of 25 µL of 11 mM D-isocitrate (58790, Merck), which gave a final concentration of 1 mM. The conversion of α-ketoglutarate (AKG) to D-isocitrate in the reductive carboxylation reaction contained 250 mM Tris HCl pH 7.6, 25 mM NADH (N8129, MiIliporeSigma, St. Louis, MO, 63103, USA) ), 5 mM MgCl_2_, 40 mM NaHCO_3_. The reaction was started with the addition of 25 µL of 11 mM α-ketoglutarate (75890, Merck), which gave a final concentration of 1 mM.

NADP+-specific isocitrate dehydrogenase (Idp1, Idp2, Idp3, EC 1.1.1.42) activity was measured according to Contreras-Shannon et al. [71]. The assay mixture contained 40 mM NADP+ (N5755, MilliporeSigma, St. Louis, MO, 63103 USA), 50 mM potassium phosphate buffer pH 7.75, 5 mM MgCl_2_ and cell free extract. The reaction was started with an addition of 25 µL of 11 mM D-isocitrate (58790, Merck) to give a final concentration of 1 mM. The conversion of α-ketoglutarate (AKG) to D-isocitrate in reduction carboxylation reaction by Idp1-3 was measured according to the protocol proposed by Contreras-Shannon et al. [71] (to Kinetic Properties and Metabolic Contributions of Yeast Mitochondrial and Cytosolic NADP+-specific Isocitrate Dehydrogenases). The reductive carboxylation reaction contained 50 mM potassium phosphate buffer pH 6.5, 0.5 mM NADPH (481,973, Merck), 5 mM MgCl_2_, 40 mM NaHCO_3_. The reaction was started with the addition of 25 µL of 11 mM α-ketoglutarate (75890, Merck), which gave a final concentration of 1 mM.

Phosphoenolpyruvate carboxykinase (Pck1, EC 4.1.1.49) activity was measured according to Roja et al. [72]. The assay mixture contained 100 mM HEPES buffer pH 7.5, 0.4 mM NADH (N8129, MilliporeSigma, St. Louis, MO, 63103, USA), 1 mM GTP (G8877, Merck), 1 mM ADP (A2754, MilliporeSigma, St. Louis, MO, 63103, USA), 4 mM MgCl_2_, 0.2 mM MnCl_2_, 0.5 U pyruvate kinase (PK-RO, Roche), 0.5 U lactic dehydrogenase (10127876001, Roche, Basel, Switzerland) and a cell-free extract. The reaction was started with the addition of 25 µL of 4.4 mM oxaloacetic acid (OAA, O4126, Merck) to produce a final concentration of 0.4 mM.

Malate dehydrogenase (Mdh1, Mdh2, Mdh3, EC 1.1.1.37) activity was measured according to Lee et al. 2009 [73]. The assay mixture contained 100 mM potassium phosphate buffer pH 7.4, 3.75 mM NADH (N8129, MilliporeSigma, St. Louis, MO, 63103, USA) and a cell-free extract. The reaction was started with the addition of 25 µL of 66 mM oxaloacetic acid (OAA, O4126, Merck) to create a final concentration of 6 mM.

NADP+-dependent malic enzyme (Mae1, EC 1.1.1.38) activity was measured according to Boles and co-authors [74]. The assay mixture contained 100 mM Tris-HCl pH 7.5, 10 mM MgCl_2_, 0.4 mM NADP+ (N5755, MilliporeSigma, St. Louis, MO, 63103, USA) and a cell-free extract. The reaction was started with an addition of 25 µL of 110 mM L-malate (M1000, Merck) to give a final concentration of 10 mM.

All assays were performed for at least three independent biological replicates. Each biological sample was measured twice in four dilutions. The presented results are the mean values with the standard deviations obtained from technical and biological replicates. Statistical significance was calculated according to Student’s *t*-test.

### 4.5. Acetyl-CoA, NAD+ and NADH Measurement

To determine acetyl-CoA levels, yeast cell extracts were prepared according to Liu, Zhang and Jiang [75]. The amount of acetyl-CoA was determined using the MAK039-1KT fluorometric kit (MilliporeSigma, St. Louis, MO, 63103, USA), according to the manufacturer’s instructions. The Acetyl-CoA concentration, expressed in ng, was calculated from a standard curve and normalized to the cells dry weight expressed in g. NAD+ and NADH levels were measured according to the NAD+/NADH Assay Kit II (colorimetric, ab221821, Abcam plc, UK) as stated in the manufacturer’s protocol. NAD+ and NADH concentrations expressed in µM were calculated from a standard curve and normalized to the total protein concentrations, expressed in mg. The assays were performed for at least three independent biological replicates.

### 4.6. Lipid Extraction and Staining

The extraction and staining of total cellular lipids was done as described by [76,77]. Briefly, 50 mL of yeast cells grown to OD600 ≈ 1.0 either in YPD or YPGly at 30 °C or 37 °C were harvested by centrifugation, lyophilized and weighed to determine dry cell weight. The lipids were extracted from the lyophilized biomass with a chloroform:methanol mixture (2:1, *v*/*v*) with Mini-Beadbeater 24 (Biospec products) using 200 μL of glass beads (425–600 μm; G8772, Sigma-Aldrich, St. Louis, MO, 63103, USA) in 8 bursts of 10 s each with breaks between the cycles for 1 min incubation on ice. Lipids were recovered by evaporation of the bottom layer solvent and then the pellet was weighed. The obtained result is the percentage of lipid content in cell dry weight.

## 5. Conclusions 

Taking into account the importance of mitochondrial activity to cellular metabolism, redox state, ATP production, and genome stability (thus longevity regulation) [78], our comprehensive ^13^C flux data on cells representing non-optimal RNAP III activity (*maf1Δ* and *rpc128-1007*) provide valuable, new insights into possible perturbations to cellular metabolism caused by the non-optimal, non-coding RNA biosynthesis [16]. This brings both detrimental consequences, which can be phenotypically observed as a growth arrest, as well as hidden metabolic changes, which in the case of unicellular eukaryotic organisms such as *S. cerevisiae* can be easily followed utilizing methods such as ^13^C flux analysis. Global metabolomics data are still difficult to interpret, for instance when performed under semi-controlled nutritional conditions. Therefore, we believe that the ^13^C flux data presented here and obtained by direct tracking of the intracellular carbon flux distribution in a living cell are of high importance and a meaningful contribution to the current state of knowledge on RNAP III global impact on metabolism of eukaryotic cells.

## Data Availability

The data presented in this study are available.

## Supplementary Materials

The following supporting information can be downloaded at: https://www.mdpi.com/article/10.3390/ijms241914763/s1.
