# Supplementary material for: Mitochondrial Metabolism in the Spotlight: Maintaining Balanced RNAP III Activity Ensures Cellular Homeostasis
Source: Int J Mol Sci. 2023 Sep 29;24(19):14763. doi: 10.3390/ijms241914763 (PMC10572830; doi:10.3390/ijms241914763)

Supplementary data

Table S1. The amino acids isotopomers percentage (%) pool in the wild-type (WT), *maf1-Δ* and *rpc128-1007* yeast cells grown in a rich medium supplemented with either 2 % [1,2-^{13}C]-glucose (A, B) or 2 % [U-^{13}C]-glycerol (C) after a temperature shift to 37°C for 1 h (D). Yeast overnight (o/n) cultures were pre-cultivated in YPGly medium (B, C, D). For the glucose labelling experiment of *maf1-Δ* and reference yeast strain (WT), overnight cells cultures were grown in YPD medium (A). The results are shown as an average percentage value from three independent biological replicates (mean) with standard deviations (SD). Zero stands for the incorporation of ^{12}C and 1 indicates ^{13}C-labelling.

A.

**2 % glucose (o/n culture 2 % glucose)**					
Amino acid	Isotopomer	WT		*maf1-Δ*	
		Mean [%]	SD	Mean [%]	SD
Alanine	0 0 0	89,20	0.81	89,82	3.0
	0 1 1	10.15	0.82	9.19	2.5
	0 0 1	0.63	0.25	0.97	0.46
Glutamate	0 0 0 0 0	82.60	0.28	83.30	0.30
	0 0 0 1 1	7.15	0.05	6.94	0.10
	1 1 0 0 0	0.30	0.01	0.37	0.05
	0 0 1 0 0	0,06	0.00	0.35	0.04
	0 1 1 0 0	7.46	0.18	6.72	0.08
	0 1 1 1 1	2.44	0.02	2.30	0.03
Aspartate	0 0 0 0	69.78	5.36	73.14	3.39
	0 0 1 1	0.0085	0.01	0.012	0.0
	0 1 1 0	34.09	5.35	26.84	3.39

B.

**2 % glucose (o/n culture 2 % glycerol)**					
Amino acid	Isotopomer	WT		*rpc128-1007*	
		Mean [%]	SD	Mean [%]	SD
Alanine	0 0 0	93.63	1.54	90.30	1.30
	0 1 1	5.75	1.28	8.61	0.95
	0 0 1	0.61	0.27	1.08	0.37
Glutamate	0 0 0 0 0	81.92	0.18	84,70	0.46
	0 0 0 1 1 1	8,09	0.48	6.76	0,33
	1 1 0 0 0	0.95	0.06	0.32	0.04
	0 0 1 0 0	0.59	0.11	0.00	0.00
	0 1 1 0	6.34	0.27	6.11	0.10
	0 1 1 1 1	2.08	0.16	2.08	0.09
Aspartate	0 0 0 0	78,69	3.38	77,86	0.80
	0 0 1 1	0.05	0.0	0.08	0.03
	0 1 1 0	21.29	3.36	22.05	0.15

C.

2 % glycerol (o/n culture 2 % glycerol)							
Amino acid	Isotopomer	WT		*maf1-Δ*		*rpc128-1007*	
		Mean [%]	SD	Mean [%]	SD	Mean [%]	SD
Alanine	0 0 0	73.60	5.94	76.33	0.29	68,63	9.45
	1 1 1	23.91	5.32	19.63	8.59	29.13	8.59
	1 1 0	0.25	0.054	0.79	0.00	0.39	0.16
	1 1 0	0.42	0.09	1.38	0.28	0.50	0.11
	0 0 1	1.80	0.47	1.84	0.14	1.98	1.19
Glutamate	0 0 0 0 0	77.95	1.42	88,24	0.19	76.79	1.32
	0 0 0 1 1	6.57	0.59	5.37	0.17	6.63	0.26
	1 1 0 0 0	5.67	0.44	3.59	0.25	6.52	0.37
	0 0 1 0 0	1.15	0.12	0.22	0.11	1.07	0.25
	1 1 1 0 0	5.58	0.58	1.76	0.14	5.24	0.28
	0 1 1 1 1	2.01	0.58	0.76	0.04	2.04	0.12
	1 1 1 1 1	1.47	0.48	0.00	0.00	1.63	0.06
Aspartate	0 0 0 0	89.30	2.27	88.69	4.59	92.68	1.89
	1 1 0 0	2.69	1.27	7.74	0.85	2.39	1.60
	0 0 1 1	2.91	1.08	3.15	0.63	1.61	0.08
	1 1 1 0	2.59	0.61	1.14	0.17	1.38	0.30
	0 1 1 1	0.61	0.55	1.30	0.23	1.92	0.58

D.

2 % glycerol, 1 h shift to 37°C (o/n culture 2 % glycerol)					
Amino acid	Isotopomer	WT		*maf1-Δ*	
		Mean [%]	SD	Mean [%]	SD
Glutamate	0 0 0 0 0	70.85	0.83	81.97	0.50
	0 0 0 1 1	8.42	0.33	6.86	0.62
	1 1 0 0 0	6.93	0.27	4.62	0.53
	0 0 1 0 0	1.33	0.37	1.56	0.52
	1 1 1 0 0	7.18	0.28	1.96	1.09
	0 1 1 1 1	2.91	0.12	1.38	0.21
	1 1 1 1 1	2.35	0.10	1.03	0.50
Aspartate	0 0 0 0	82.03	1.52	88.04	0.83
	1 1 0 0	4.98	1.03	4.35	0.76
	0 0 1 1	4.45	0.18	3.82	0.46
	1 1 1 0	4.88	0.19	2.14	0.47
	0 1 1 1	3.62	0.14	1.63	0.16

Table S2. Reaction fluxes modified in delta_maf1FBA model. Abbreviations: LB- lower bound, UB- upper bound

Reaction ID	LB	UB	Gene	Function
R_04490.0	0.0	0.1	FBP1	Fructose-1,6-bisphosphatase, key regulatory enzyme in gluconeogenesis
R_11340.0	0.0	0.1	HXT6&7	High affinity glucose transporter
R_11390.0	0.0	0.1	HXT6&7	High affinity glucose transporter
R_11660.0	0.0	0.1	HXT6&7	High affinity glucose transporter
R_09890.0	0.0	0.1	LYS9	Saccharopine dehydrogenase, the seventh step in lysine biosynthesis, exhibits genetic and physical interaction with TRM12 (involved in tRNA methylation)
R_05490.0	0.0	0.1	MET2	L-homoserine-O-acetyltransferase, the first step of the methionine biosynthesis
R_08840.0	0.0	0.1	PCK1	Phosphoenolopyruvate carboxykinase, key enzyme in gluconeogenesis

Table S3. FVA and FBA simulation results indicating increased activity of Pda1 (the PDH complex subunit) in delta_maf1FBA model. Delta_maf1FBA model represents *maf1Δ* metabolism, the consensus Yeast7.6 model represents metabolism of the WT strain. Abbreviations: LB- lower bound, UB- upper bound, FBA- Flux Balance Analysis, FVA- Flux Variability Analysis.

**strain**	**Reaction in the model**	**LB**	**UB**
glucose			
WT	R_PDA1	0.0	4.0
maf1Δ	R_PDA1	0.0	3.07831
glycerol			
WT	R_PDA1	0.0	2.0
maf1Δ	R_PDA1	0.0	0.929155
galactose			
WT	R_PDA1	0.0	4.0
maf1Δ	R_PDA1	0.0	3.07831

**strain**	**Reaction in the model**	**FBA**
glucose		
WT	R_PDA1	2.01943e-13
maf1Δ	R_PDA1	1.67712
glycerol		
WT	R_PDA1	0.0
maf1Δ	R_PDA1	0.755131
galactose		
WT	R_PDA1	0.0
maf1Δ	R_PDA1	1.75513

Figure S1. Lipid droplets in the strains.

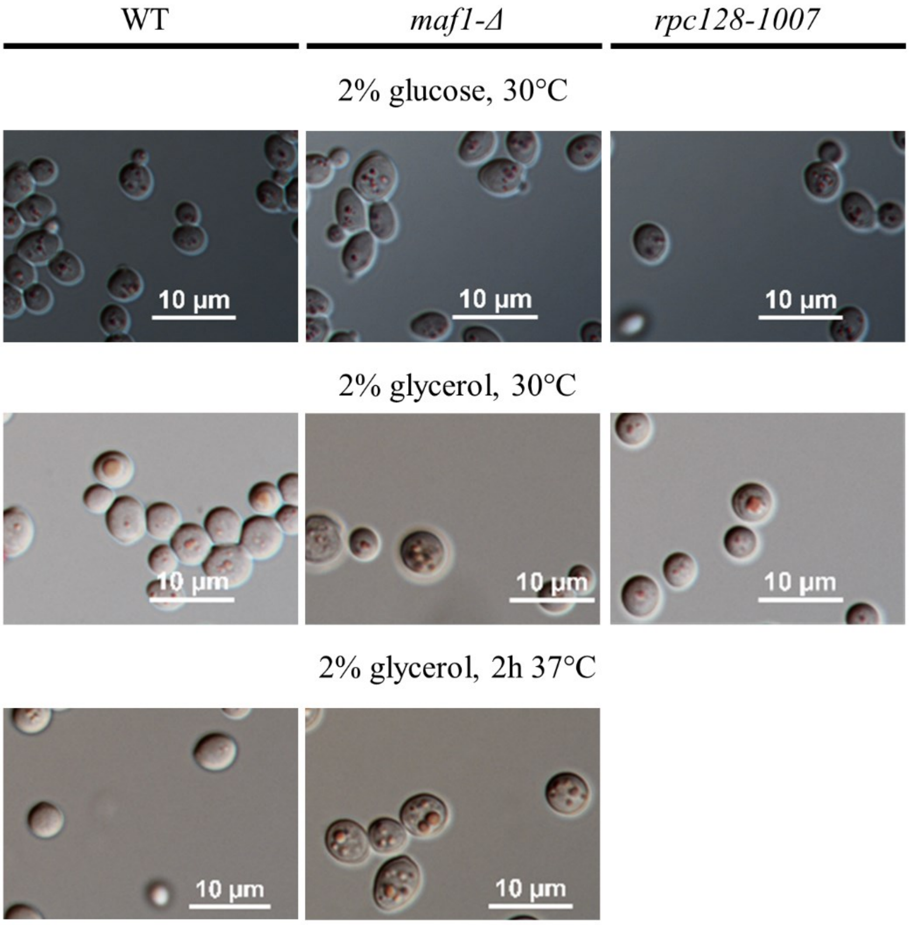

Supplement: Supplementary file 1 [file ijms-24-14763-s001.zip › ijms-2542961-supplementary.pdf]
